# Academic Primer Series: Five Key Papers Fostering Educational Scholarship in Junior Academic Faculty

**DOI:** 10.5811/westjem.2016.7.31126

**Published:** 2016-08-22

**Authors:** Teresa M. Chan, Michael Gottlieb, Abra L. Fant, Anne Messman, Daniel W. Robinson, Robert R. Cooney, Dimitrios Papanagnou, Lalena M. Yarris

**Affiliations:** * McMaster University, Department of Medicine, Division of Emergency Medicine, Hamilton, Ontario, Canada; † Rush University Medical Center, Department of Emergency Medicine, Chicago, Illinois; ‡ Northwestern Memorial Hospital, Department of Emergency Medicine, Chicago, Illinois; § Sinai Grace Hospital, Department of Emergency Medicine, Detroit, Michigan; ¶ University of Illinois, Department of Emergency Medicine, Chicago, Illinois; || Geisinger Medical Center, Department of Emergency Medicine, Danville, Pennsylvania; # Sidney Kimmel Medical College, Thomas Jefferson University, Department of Emergency Medicine, Philadelphia, Pennsylvania; ** Oregon Health & Science University, Department of Emergency Medicine, Portland, Oregon

## Abstract

**Introduction:**

Scholarship is an essential part of academic success. Junior faculty members are often unfamiliar with the grounding literature that defines educational scholarship. In this article, the authors aim to summarize five key papers which outline education scholarship in the setting of academic contributions for emerging clinician educators.

**Methods:**

The authors conducted a consensus-building process to generate a list of key papers that describe the importance and significance of academic scholarship, informed by social media sources. They then used a three-round voting methodology, akin to a Delphi study, to determine the most useful papers.

**Results:**

A summary of the five most important papers on the topic of academic scholarship, as determined by this mixed group of junior faculty members and faculty developers, is presented in this paper. These authors subsequently wrote a summary of these five papers and discussed their relevance to both junior faculty members and faculty developers.

**Conclusion:**

Five papers on education scholarship, deemed essential by the authors’ consensus process, are presented in this paper. These papers may help provide the foundational background to help junior faculty members gain a grasp of the academic scholarly environment. This list may also inform senior faculty and faculty developers on the needs of junior educators in the nascent stages of their careers.

## INTRODUCTION

Academic scholarship is often intimidating for junior clinician educators. While scholarship is a time-honored tradition within residency programs, success during residency training may not appropriately prepare graduating residents for the challenges and expected benchmarks for a profession in academic medicine. Junior (and even mid-level) faculty members may encounter challenges in finding successful academic direction at their respective institutions.^1^–^5^ Junior faculty members newly immersed in an academic institution may face competing demands for clinical productivity; teaching and mentoring students and residents; securing funding for and engaging in research; and finding harmony with work-life balance.^5^ The aforementioned challenges have the potential to undermine junior faculty members’ confidence. If these faculty members are not nurtured in the nascent stages of their careers and do not receive the necessary mentorship and institutional resources that are essential for their development, their respective trajectories as mid-level faculty may be stunted.^4^

Discovering an academic niche and building a portfolio provides professional stability and supports academic appointments and promotions. Despite a track record of scholarly, evidence-based teaching, most tenure and promotion committees reward academically-sound, scholarly productivity more substantively. When considering candidates for promotion to assistant, associate, and full professor, a track record of peer-reviewed scholarship justifies the committee’s decision.

Clinician educators may feel isolated, and left to fend for themselves among more senior core faculty. Those who are interested in educational or academic scholarship are often viewed differently from their research colleagues. This can make success as an academic clinician educator challenging.

The Academic Life in Emergency Medicine (ALiEM) Faculty Incubator was created in 2016 to address several of these issues. During our one-year experience, we created modules in which we described and discussed key literature relevant to junior clinician educators embarking on their respective careers within academic medicine. Our paper is a synthetic, narrative review that highlights some of the most important literature on the topic of academic scholarship, the first topic covered in our discovery-based Faculty Incubator curriculum.

## METHODS

In the first month of the ALiEM Faculty Incubator, we discussed the topic of educational scholarship. We allowed the discussion to unfold, as we gathered the titles of papers that were cited, shared, suggested, and discussed within the online discussion forum. This forum consists of 30 junior faculty members and eight facilitators (faculty mentors and administrators) that exists via a closed, mixed-media, social media platform (Slack.com, San Francisco, CA). This platform allows for text-based communication, augmented by file-sharing and embedded website links. The discussion that occurred involved an international group of clinician educators spanning three countries (e.g. United States, Canada, and Chile) and multiple time zones.

We monitored the proceedings of the ALiEM Faculty Incubator from March 1–31, 2016, during which time all members participated asynchronously in various discussions around the topic of academic scholarship.

The list generated by the Faculty Incubator proceedings was then supplemented with a general call for suggestions from several social media outlets to optimize the literature list. On Twitter, we “tweeted” requests to have participants of the #FOAMed and #MedEd online communities provide suggestions for important papers on the topic of educational scholarship within emergency medicine (EM). The figure shows an exemplar request tweet.

Once our list of the most high-yield papers on educational scholarship was compiled, the authors subsequently conducted a three-round voting process, inspired by the Delphi methodology. We have not described our method as a pure Delphi methodology since our authorship panel comprises both novices (i.e. junior faculty members, participants in the Faculty Incubator) and experts in the field (i.e. experienced clinician educators, all of whom have published >10 peer-reviewed publications, who serve as mentors and facilitators of the Faculty Incubator). The selection of articles by both novices and experts was intentional: the authors sought to find articles that would both meet the approval of experienced clinician educators and resonate with junior faculty members entering the field of academic medicine. The rationale for reducing the number of papers to five was in an effort to restrict the list to the most crucial papers for junior clinicians to read, and subsequently to provide a short synopsis to explain to both junior educators and faculty developers why these papers were thought to be highly relevant. Of note, we have provided the complete list of all relevant papers discussed in these proceedings in the Table (first column) for those interested in an expanded reading list.

## RESULTS

Our initial review of the ALiEM Faculty Incubator discussion thread yielded a total of 17 articles, which were mentioned by mentors and the junior faculty incubator participants (heretofore dubbed *incubatees*). The social media calls over one week (March 18–25, 2016) yielded eight additional suggested articles leading to a total of 25 articles for evaluation by our team. The three-round voting procedure allowed our team to generate a rank-order listing of all these papers in order of relevance, from the most important to the least important. The citations and our ratings of these 25 papers are listed in the Table.

## DISCUSSION

The following are summaries of the top five papers accompanied with commentaries on their relevance to both junior faculty members, as well as potential considerations for faculty developers when discussing these works.

### 1. Beckman TJ, Cook DA. Developing scholarly projects in education: a primer for medical teachers*. Med Teach*. 2007 Mar;29(2–3):210–8.^6^

#### Summary

In academic medicine, promotion is often based on productivity, which in many universities is synonymous with publication. Publication within medical education, however, is often difficult given the various approaches to study design that are unique to the field and disparate from clinical medicine. This paper outlines a three-step method to rigorously develop scholarly education projects that can potentially lead to peer-reviewed publication.^6^ Step 1 is to *Refine the Study Question*.^6^ This involves a rigorous review of the current literature by querying traditional and alternative databases, as well as using specific search terms and canvassing the bibliographies of selected articles.^6^ This step also involves composing a clear and succinct problem statement that navigates from existing knowledge to the new information the project will contribute to the literature. Initial refinement, per Beckman and Cook, requires the incorporation of a conceptual framework to provide a theoretical context. Finally, the authors suggest that the investigator generate a statement of study intent that includes a hypothesis, where appropriate.^6^ Step 2 is to *Identify Study Design and Method*.^6^ Here the paper gives a brief overview of different study designs applicable to medical education scholarship. Lastly, Step 3 is to *Select Outcomes,* which involves selecting outcomes that balance feasibility and meaningfulness according to Kirkpatrick’s Hierarchy.^6^ By following these steps, a medical educator can design an effective and publishable scholarly education project.^6^

#### Relevance to Junior Faculty Members

This is an essential primer for junior faculty members, especially for those who lack direct mentorship or prior training in education scholarship. The “publish or perish” paradigm is still part of the cultural milieu in many university settings. It is, therefore, important for junior faculty members to obtain academic credit for their educational work via traditional, peer-reviewed publication, commonly via program evaluation as suggested by this paper. While most trainees and junior faculty members are exposed to research design, there are subtleties and differences to education scholarship that are not intuitive for newcomers to the field. In order to perform education scholarship in a meaningful and effective way, junior faculty members need faculty development. This paper provides a foundation for this design, and offers the junior faculty educator a starting point for further learning.

#### Considerations for Faculty Developers

Faculty developers will find this primer provides helpful information to teach three key groups: 1) educators interested in scholarship, 2) clinical researchers without education expertise, and 3) scholars who are interested in building skills as peer reviewers. Not only does it provide a general overview of the landscape of education scholarship, but it also provides readers with concrete steps for program evaluation. Written from the perspective of an education scholar, this piece highlights the concepts that are of unique importance in medical education, such as the development of a conceptual framework; education-specific study designs (i.e., validity studies); and selecting relevant outcomes for educational studies. A potential point of confusion in this paper is Step 3, which places a significant focus on the Kirkpatrick Model for outcome measures. When using this paper, it may be prudent to discuss outcome measures with junior colleagues beyond this model. Overall, the references cited include a good starting bibliography for education scholars.

### 2. Yarris LM, Deiorio NM. Education research: a primer for educators in emergency medicine. *Acad Emerg Med*. 2011 Oct;18 Suppl 2:S27–35

#### Summary

Yarris and Deiorio lay out a broad overview of educational research in EM for new educational researchers.^7^ They formulate one approach for conducting high quality research in education.^7^ The general steps are as follows: 1) Identify a research problem; 2) Perform a literature review with annotated bibliography; 3) Identify a conceptual framework to frame the problem and research question; 4) Craft a research question using the *FINER* (Feasible, Interesting, Novel, Ethical, and Relevant) approach; 5) Select a study design; 6) Conduct the research using your well developed research plan; and 7) Dissemination of said research.^7^ The authors pay particular attention to details of how to perform each step, especially focusing on qualitative and quantitative study designs and how they are important, equal, and valid types of data.^7^ At the end of the paper the authors present the common barriers and solutions new researchers must overcome to perform such research.^7^ Yarris and Deiorio provide further reading on specific topics, as well as advice to the early-career education researcher.^7^

#### Relevance to Junior Faculty Members

Being a junior academic faculty member can be a stressful experience secondary to the demands on publishing, as has been previously mentioned. Applying perspectives from Yarris and Deiorio’s paper, however, can make this period far less stressful. Their paper offers a general framework for how to approach research from the outset to ensure high quality, publishable research.

Novel to this paper is its emphasis on methods and study design, and more specifically the differences between qualitative and quantitative research. Qualitative methodologies are particularly important in educational research, and are poorly taught compared to more traditional quantitative methodologies seen in healthcare research. The paper also impresses upon junior faculty members to ensure that they move beyond measuring Kirkpatrick Level 1 type outcomes (i.e. “I liked this” or “I didn’t like this”).^7^

#### Considerations for Faculty Developers

In 1990, Boyer expanded the definition of scholarship to include the scholarship of teaching and learning (SoTL).^30^ Clinician-educators are in an ideal position to engage in SoTL, though many lack experience in conducting educational research. This article reviews the process of engaging in educational scholarship and research, providing a structure that faculty developers will find helpful in mentoring junior faculty. While some educational research methodologies may overlap with traditional biomedical research, other methodologies, such as qualitative research techniques, are more likely to be used in educational or social science research. Furthermore, traditional EM specialty journals may not be as receptive to educational research. Educational scholars will find the section on dissemination of educational research helpful in pursuing publication for their manuscripts.

### 3. Bandiera G, Lee S, Tiberius R. Creating effective learning in today’s emergency departments: how accomplished teachers get it done. *Ann Emerg Med*. 2005 Mar;45(3):253–61.^8^

#### Summary

While there is a fair amount of research on clinical teaching and on the qualities of a successful clinical teacher, very little research focuses on clinical teaching in the emergency department (ED). Clinical teaching in the ED has its unique challenges and opportunities that require specific strategies for success. Bandiera et al. interviewed successful clinical teachers in the ED to ascertain behaviors that make these teachers successful.^8^ Using a qualitative methodology, interviews were analyzed using a rigorous method with multiple coders.^8^ From this analysis, 12 specific strategies were identified that any clinical teacher can use. These 12 strategies are explored in this paper with specific examples of their implementation provided.^8^ Perceived barriers to excellent clinical teaching are also discussed.^8^ Upon reading this paper, readers can expect to have a concrete idea of how they can improve their clinical teaching abilities on their next clinical shift and beyond.

#### Relevance to Junior Faculty Members

What makes a successful clinical teacher in the ED? This article answers that question succinctly with realistic strategies any clinical educator can implement. Most junior faculty members have a strong desire to be excellent clinical teachers, but few will know how to accomplish this. Bandiera et al. went directly to the source – excellent clinical teachers – and asked them what behaviors and attributes make them great teachers.^8^ Twelve themes emerged from these interviews. Junior faculty will find this article invaluable in shaping their teaching strategies in the ED despite the threats of time constraints and lack of interest. Junior faculty will learn how to effectively teach learners of all levels of training, including non-EM learners rotating in the ED.

#### Considerations for Faculty Developers

While interested junior faculty members can attend general faculty development programming within the affiliated medical school that provides their faculty appointment, it is essential that academic EM departments offer specialty-specific faculty development programming. Junior faculty members will require the skill set to teach trainees in the clinical learning environment. This environment, however, can vary from a controlled setting conducive to bedside teaching, to one replete with chaos and multiple resuscitations. To be successful in either milieu, junior faculty will need a checklist of basic educational practices that can guide and facilitate their instruction of trainees.

This article provides the ideal framework for the faculty developer. Each item on the checklist featured in this paper can be developed into its own mini-workshop or lecture, thereby providing the faculty developer with a curriculum for the content with which the junior faculty should become acquainted. The practices discussed here will refresh and reinvigorate faculty who may have adopted a routine practice of teaching, and allow for the reflection of best practices to incorporate in their repertoire of teaching in the ED. Most interestingly, this paper’s list of barriers to good ED teaching is a good reminder that all of us face the same problems, whether we are junior educators or senior, award-winning faculty members.

On another level, this paper also serves as an example of a paper using a qualitative methodology to answer a research question. This paper can be used by faculty developers to teach the critical appraisal of qualitative studies, as it offers a variety of excellent points for discussion (e.g. multiple coders).

### 4. Schrager S, Sadowski E. Getting More Done: Strategies to Increase Scholarly Productivity. *J Grad Med Educ*. 2016 Feb;8(1):10–3.^9^

#### Summary

This is a narrative review paper that aggregates commonly provided advice and strategies for junior scholars who are interested in being more productive. Advice given covers topics such as the following: 1) The “To do” list; 2) Finding a Balance: Learning to Say “No”; 3) Increasing Productivity by Making Everything Count Twice; and 4) Being Efficient.^9^

#### Relevance to Junior Faculty Members

Junior faculty are often pulled in many directions, expected to balance teaching, research, mentoring, and clinical work. It can be challenging to balance all of these demands while also maintaining wellness. This paper provides a variety of strategies for selecting projects that align more with personal goals, as well as learning how to say “no.”^9^ It can be challenging to decline projects when beginning an academic career, but one must also consider the opportunity costs associated with every new project. Additionally, the authors describe strategies for maximizing the value in projects. Examples include converting a new curricular change into a research project and publication, or using the knowledge obtained from a literature review to develop a grand rounds or national lecture.^9^ Finally, the authors emphasize the importance of avoiding distractions and various techniques for how to minimize them.^9^ Strategies include targeted email checking, scheduling meetings in blocks, techniques for reducing procrastination, and avoiding time wasted on excessive perfectionism (which can be a form of procrastination).^9^

#### Considerations for Faculty Developers

Academic productivity is not exclusive to junior attendings in EM. Interestingly, the paper cites very diverse sources of these insights ranging from *Forbes* magazine to *Psychology Today* to academic blogs, suggesting that perhaps sometimes the answers we seek may not be simply accessible through PubMed. That said, in EM we are fortunate to have tacit wisdom available via our own academic blogs. For faculty developers looking for more insight on this topic, we suggest you might augment your teaching and reading with the ALiEM *How I Work Smarter* series (http://www.aliem.com/category/non-clinical/how-i-work-smarter/).

### 5. Cristancho S, Varpio L. Twelve tips for early career medical educators*. Med Teach*. 2015 Oct 22:1–6. [Epub ahead of print]^10^

#### Summary

This is a paper from the *12 Tips* series published in *Medical Teacher*. It contains excellent suggestions and tips gathered and then written by early-career medical educators in Canada.^10^ Tips range from advice on how to think and talk about oneself (Tip 1: Articulate your area(s) of interest; Tip 8: Create Multiple Elevator Pitches; Tip 11: Embrace your identity within the field) to “nuts and bolts” ideas (Tip 4: Develop strong communication skills; Tip 5: Cultivate relationships with mentors; Tip 6: Be a good mentee; Tip 10: Build resilience as your armor).^10^

#### Relevance to Junior Faculty Members

This article provides advice and concrete tips for developing both short- and long-term career plans, and how to align one’s goals with that of the institution.^10^ Junior faculty members will benefit from understanding how to better align their projects to avoid being spread too thin, with a focus on longer-term goals to support both career and life satisfaction.^10^

This article also highlights the interconnectedness of the medical education community, emphasizing the importance of one’s peers in an early-career educator’s career path. Often times, networking is underemphasized in medicine when compared to other industries, much to the disadvantage of junior faculty members. Effective networking can lead to new projects, more mentors, exciting job opportunities, and letters from other academics in the field, which are required for promotions.^10^ The authors stress the importance of mentorship along with strategies for effectively selecting a mentor and being a good mentee.^10^ Regrettably, the only thing the authors did not provide insight on was how to be a good mentor in turn.

#### Considerations for Faculty Developers

This paper reminds us that medical education is a field (Tip 11), wherein many people of various backgrounds come together to work in the same arena – a concept that is important to explain when orienting junior faculty members.^10^ Understanding this concept early allows junior faculty members to better understand why there are pluralistic views and perspectives within medical education as manifest via presentations, papers, and even reviews of their scholarship. For those developers who wish to have an EM specialty-specific bend on the topic of networking, another article has recently been published on this topic.^31^

#### Honorable Mention

##### Sherbino J, Arora VM, Van Melle E, Rogers R, Frank JR, Holmboe ES. Criteria for social media-based scholarship in health professions education. *Postgrad Med J*. 2015 Oct;91(1080):551–5.^11^

Although it was not in the top five papers, this paper was quite highly valued by our panel. The age of social media is upon us, and in EM it is especially important for junior faculty members to be aware of how social media-based work might be turned into scholarship. However, since within many academic environments this is not yet a standard form of scholarship, we were reluctant to endorse it as one of the top five papers. Some departments and universities are beginning to consider the impact of such scholarly works within their academic promotions and tenure process.^32^ If a junior faculty member is hired into environments that are supportive of digital and social media scholarship, this paper will be essential for guiding them towards a scholarly approach for their work. For more senior faculty members, this may be a paper that is worth keeping in your reading file to use when you want to support a junior faculty member doing great work, or maybe if you are looking to pave the way towards a more broadened definition of scholarship at your institution.

## LIMITATIONS

The main limitation of our proceedings is that our search strategy was not comprehensive. Although we attempted to gather recommendations from multiple sources (e.g., our Faculty Incubator discussions, Twitter), we did not perform an exhaustive, structured literature review. The purpose of this paper, however, was to aggregate an approachable set of high-yield papers that will serve as a starting point for junior faculty members embarking on their academic EM careers. The authors hope that this is the starting point for their exploration and initial development.

## CONCLUSION

We have provided a reading list that may be beneficial as an introduction for junior faculty members to the world of educational scholarship. We hope this paper provides junior clinician educators a broad overview of this important topic and makes it more approachable and less intimidating.

## Figures and Tables

**Figure:**
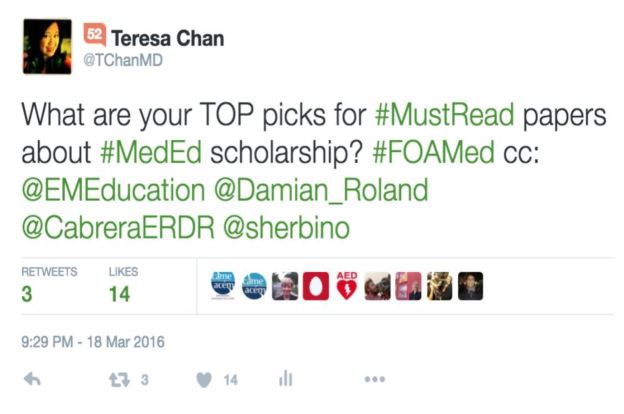
Exemplar “tweet” calling for online contributions for papers on medical education collaboration.

**Table:** The complete list of educational scholarship literature collected by the authorship team.

Citation	Round 1 initial mean scores (SD) max score 7	Round 2 % of raters that endorsed this paper	Round 3 % of raters that endorsed paper in last round	Top 5 papers
Beckman TJ, Cook DA. Developing scholarly projects in education: a primer for medical teachers. *Med Teach*. 2007 Mar;29(2–3):210–8.^6^	6.87 (0.35)	100%	100%	1
Yarris LM, Deiorio NM. Education research: a primer for educators in emergency medicine. *Acad Emerg Med*. 2011 Oct;18 Suppl 2:S27–35.^7^	6.63 (0.74)	100%	100%	2
Bandiera G, Lee S, Tiberius R. Creating effective learning in today’s emergency departments: how accomplished teachers get it done. *Ann Emerg Med*. 2005 Mar;45(3):253–61.^8^	6.00 (1.07)	75%	62.5%	4
Schrager S, Sadowski E. Getting More Done: Strategies to Increase Scholarly Productivity. *J Grad Med Educ*. 2016 Feb;8(1):10–3.^9^	5.89 (1.26)	87.5%	87.5%	3
Cristancho S, Varpio L. Twelve tips for early career medical educators. *Med Teach*. 2015 Oct 22:1–6. [Epub ahead of print]^10^	5.89 (0.99)	75%	62.5%	5
Sherbino J, Arora VM, Van Melle E, Rogers R, Frank JR, Holmboe ES. Criteria for social media-based scholarship in health professions education. *Postgrad Med J*. 2015 Oct;91(1080):551–5.^11^	5.63 (0.52)	75%	50%	Honorable Mention
Thurgur L, Bandiera G, Lee S, Tiberius R. What do emergency medicine learners want from their teachers? A multicenter focus group analysis. *Acad Emerg Med*. 2005 Sep;12(9):856–61.^12^	5.63 (0.74)	37.5%	25%	
Cook DA, West CP. Perspective: Reconsidering the focus on “outcomes research” in medical education: a cautionary note. *Acad Med.* 2013 Feb;88(2):162–7.^13^	5.25 (1.16)	37.5%	0%	
Sutkin G, Wagner E, Harris I, Schiffer R. What makes a good clinical teacher in medicine? A review of the literature. *Acad Med*. 2008 May;83(5):452–66.^14^	5.00 (1.07)	25%	0%	
Glassick CE. Boyer’s expanded definitions of scholarship, the standards for assessing scholarship, and the elusiveness of the scholarship of teaching. *Acad Med*. 2000 Sep;75(9):877–80.^15^	5.00 (1.51)	25%	0%	
Côté L, Turgeon J. Appraising qualitative research articles in medicine and medical education. *Med Teach*. 2005 Jan;27(1):71–5.^16^	4.88 (1.13)	25%	0%	
Sherbino J, Van Melle E, Bandiera G, McEwen J, Leblanc C, Bhanji F, Frank JR, Regehr G, Snell L. Education scholarship in emergency medicine part 1: innovating and improving teaching and learning. *CJEM*. 2014 May;16 Suppl 1:S1–5.^17^	4.63 (0.92)	12.5%	0%	
Sullivan GM, Sargeant J. Qualities of Qualitative Research: Part I. *J Grad Med Educ.* 2011 Dec;3(4):449–52.^18^	4.50 (0.93)	25%	0%	
Sargeant J. Qualitative Research Part II: Participants, Analysis, and Quality Assurance. *J Grad Med Educ*. 2012 Mar;4(1):1–3.^19^	4.50 (0.93)	0%	0%	
O’Brien BC, Harris IB, Beckman TJ, et al. Standards for Reporting Qualitative Research: A Synthesis of Recommendations. *Acad Med.* 2014 Sep;89(9):1245–51.^20^	4.50 (1.07)	25%	0%	
Wright S, O’Brien BC, Nimmon L, et al. Research Design Considerations. *J Grad Med Educ*. 2016 Feb;8(1):97–8.^21^	4.50 (1.51)	12.5%	0%	
Ericsson KA, Krampe RT, and Tesch-Romer C. The Role of Deliberate Practice in the Acquisition of Expert Performance. *Psychological Revi*ew. 1993; 100(3): 363–406.^22^	4.38 (0.92)	12.5%	0%	
Choo EK, Ranney ML, Chan TM, et al. Twitter as a tool for communication and knowledge exchange in academic medicine: A guide for skeptics and novices. *Med Teach*. 2015 May;37(5):411–6.^23^	4.38 (0.52)	0%	0%	
Perry M, Hopson L, House JB, et al. Model for Developing Educational Research Productivity: The Medical Education Research Group. *West J Emerg Med*. 2015 Nov;16(6):947–51.^24^	4.38 (1.68)	12.5%	12.5%	
Mays N, Pope C. Assessing quality in qualitative research. *BMJ*. 2000 Jan 1;320(7226):50–2.^25^	3.75 (1.39)	0%	0%	
Kitto SC, Chesters J, Grbich C. Quality in qualitative research. *Med J Aust*. 2008 Feb 18;188(4):243–6.^26^	3.75 (1.49)	0%	0%	
Kessler C, Burton JH. Moving Beyond Confidence and Competence: Educational Outcomes Research in Emergency Medicine. *Acad Emerg Med.* 2011 Oct;18 Suppl 2:S25–6.^27^	3.75 (1.83)	12.5%	12.5%	
Shapiro ED, Coleman DL. The scholarship of application. *Acad Med.* 2000 Sep;75(9):895–8.^28^	3.75 (1.49)	0%	0%	
Hu WC, Thistlethwaite JE, Weller J, et al. ‘It was serendipity’: a qualitative study of academic careers in medical education. *Med Educ.* 2015 Nov;49(11):1124–36.^29^	3.38 (1.30)	0%	0%	
